# Novel technique to repair a bronchopleural fistula after esophagectomy using wide intercostal muscle flap fixed by azygos vein and bronchus: a case report

**DOI:** 10.1186/s40792-020-00908-8

**Published:** 2020-07-29

**Authors:** Yohei Honda, Akihiro Taira, Ayako Hirai, Koji Kuroda, Yoshinobu Ichiki, Fumihiro Tanaka

**Affiliations:** 1 Second Department of Surgery, University of Occupational and Environmental Health, 1-1 Iseigaoka, Yahatanishi-ku, Kitakyushu, 807-8555 Japan; 2 Department of Thoracic Surgery, Shimonoseki City Hospital, 1 Chome-13-1 Koyocho, Shimonoseki, Yamaguchi, 750-0041 Japan

**Keywords:** Bronchopleural fistula, Muscle flap closure, Esophagectomy

## Abstract

**Background:**

Post-esophagectomy bronchopleural fistulas can be life-threatening in patients who are exhausted, for example, by surgical stress and pleural infection; therefore, establishment of a reliable surgical procedure is extremely important. We here report a novel procedure entailing muscle flap closure for bronchopleural fistula.

**Case presentation:**

A 64-year-old man developed a right bronchopleural fistula after esophagectomy. Because he was exhausted by surgical stress and malnourished, we considered reliable surgical closure of the fistula essential. Intraoperatively, it was found to connect with the membranous portion of the right main bronchus. We decided to close the fistula with a pedicled fourth and fifth intercostal muscle flap. After separating the intercostal muscles near the angle of the rib, we passed a muscle flap between the azygos vein and bronchus and sutured it securely to the fistula. The postoperative course was uneventful, and there was no thoracic infection. Postoperative bronchoscopy confirmed the muscle flap had securely closed the fistula.

**Conclusions:**

The route and suturing technique of the intercostal muscle flap to a fistula are important, especially in exhausted patients.

## Introduction

Bronchopleural fistulas (BPF), which are defined as direct communications between a bronchus and the pleural space [1, 2], are an infrequent but life-threatening complication of thoracic surgery [3, 4]. Muscle flap closure is an effective treatment for BPF. However, affected patients are generally exhausted by surgical stress, the BPF, and pleural infection. Additionally, the optimal surgical procedure for BPF depends on various factors, including the size and site of the fistula, nutritional condition, and comorbidities [5]. It is extremely important that the selected surgical procedure fixes the muscle flap such that it does not subsequently collapse. Esophageal cancer may be complicated by infiltration of the membranous portion into the trachea and main bronchus, putting the patient at high risk of post-resection BPF. There are few published reports on surgical techniques for treating fistulas of the membranous portion of the main bronchus. Here we report successful closure of such a fistula affecting the right main bronchus by using a unique pedicled intercostal muscle flap.

## Case presentation

**Fig. 1:**
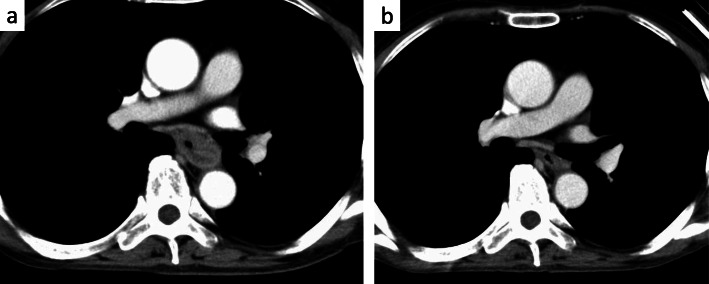
Chest CT scan showing **a** the esophageal cancer contacts to the left main bronchus and both the lower lobe of the lung before NAC. **b** After neoadjuvant chemotherapy, esophagus wall thickening had somewhat improved. However, invasion of adjacent organs could not be ruled out

**Fig. 2:**
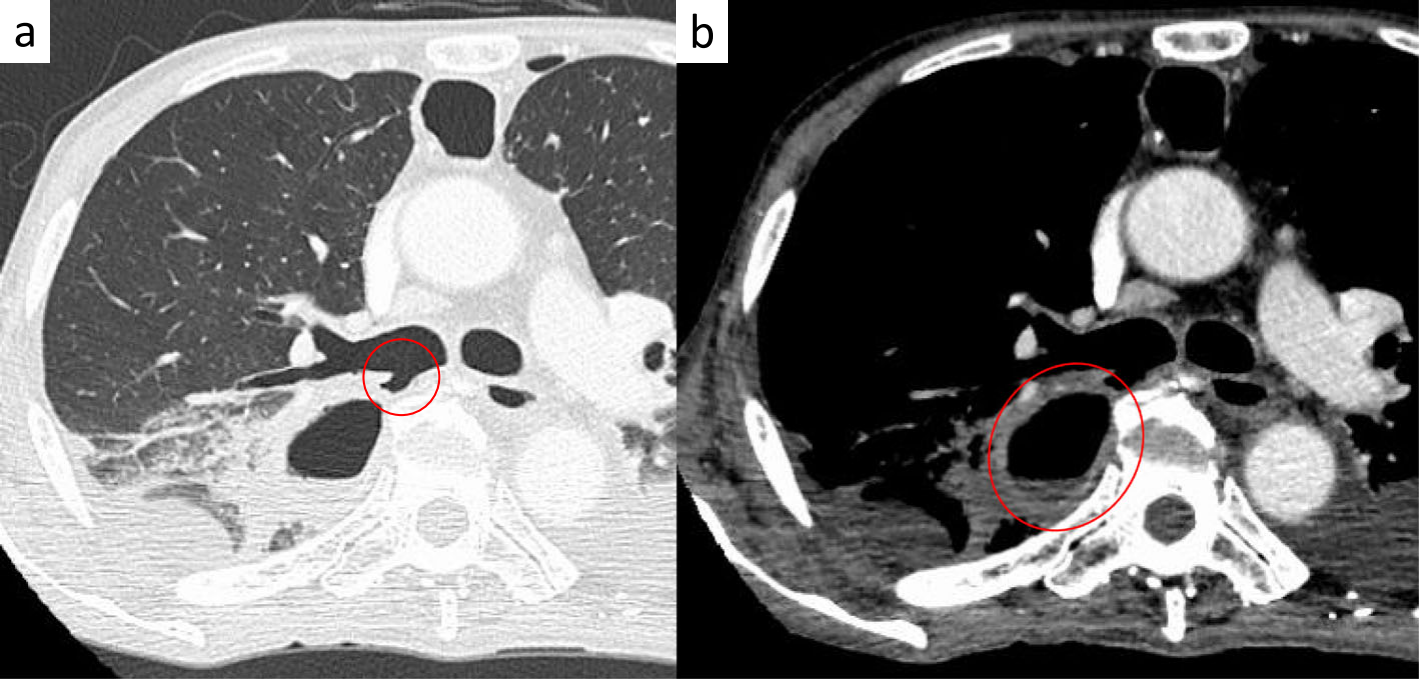
Chest CT scan images showing **a** a defect in the membranous portion of the right main bronchus and **b** an air space and air–fluid level near the right main bronchus

**Fig. 3:**
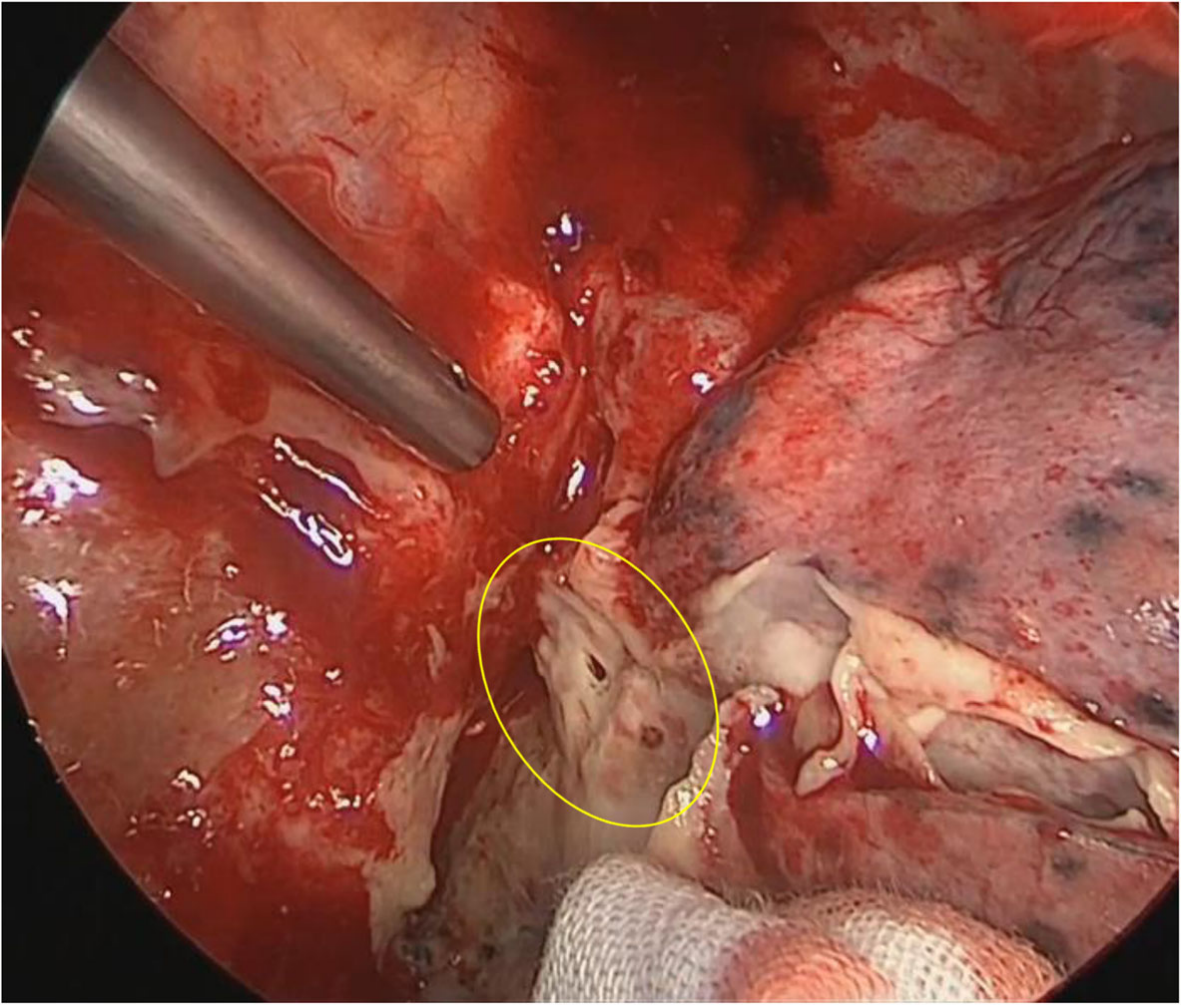
Intraoperative views showing a fistula and necrotizing changes affecting the membranous portion of the right main bronchus (inner part of the yellow circle)

**Fig. 4:**
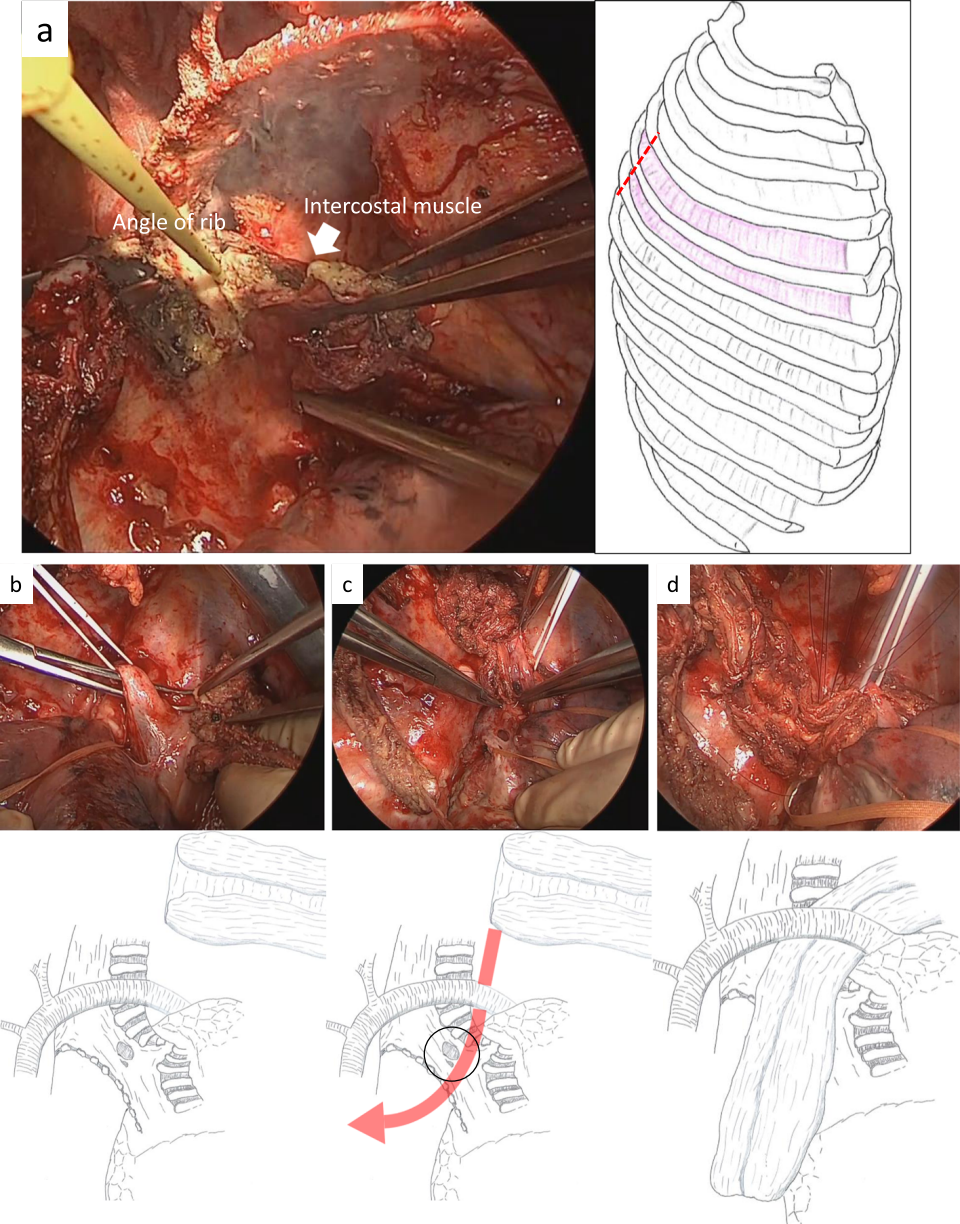
Intraoperative views showing the separation of the fourth and fifth intercostal muscles near the angles of the ribs (**a**). Next, the arrow shows the passing of the muscle flap between the azygos vein and right upper lobe bronchus. The circle indicates the anastomosis site of the bronchus and intercostal muscle flap (**b**, **c**). Post-anastomosis findings show the muscle flap covering the fistula while being supported by the right upper bronchus from below (**d**)

A 64-year-old man presented to the department of digestive surgery in our institute because of esophageal cancer in the middle and lower thoracic esophagus. Physical examination revealed severe emaciation and sarcopenia (loss of skeletal muscle volume due to malnutrition and esophageal cancer itself). The patient lost 10 kg during the last 6 months. The BMI before esophagectomy was 16.9 (height, 166.2 cm; weight, 46.7 kg). Esophageal angiography revealed an esophageal stricture across the Th6–9 levels. Furthermore, CT findings suggested that it was infiltrating both lower lungs and left primary bronchus (Fig. 1a). The upper endoscopy revealed a type 2 tumor located in the thoracic esophagus (distance from the incisors was 25 to 33 cm). The pathological diagnosis of the tumor was squamous cell carcinoma. Thus, the patient was diagnosed as having esophageal cancer cT4bN0M0 stage IIIB in accordance with the eighth edition UICC TNM classification. The patient underwent two courses of neoadjuvant chemotherapy (NAC) with docetaxel, cisplatin, and 5-fluorouracil (DCF). NAC somewhat improved esophageal wall thickening (Fig. 1b). However, the surgeons considered NAC did not achieve downstaging (tumor invasion to lungs and bronchus did not change). The patient underwent thoracoscopic subtotal esophagectomy with cervical esophagogastric anastomosis via the retrosternal route using a gastric tube. Intraoperatively, a tumor was found to be invading the right main bronchus and right lung. Wedge resection of the part of the lesion invading the lung was performed; however, resection of the part invading the bronchus was considered impossible. Therefore, LigaSure Maryland was mainly used to resect as much of the tumor as possible along the wall of the right main bronchus. After esophagectomy, the surgeons cauterized the unresectable tumor as much as possible. As a result, R2 resection was performed according to intraoperative and pathological findings. After esophagectomy, total parenteral nutrition had performed at first. Activities of daily living (ADL) of the patient was movement only in bed because he was exhausted and depressed due to surgical stress. Therefore, it was considered that sarcopenia had deteriorated. Postoperative day (POD) 6 after esophagectomy, the patient developed a fever. A partial defect of the membranous portion of the right main bronchus and an air–fluid level around the right main bronchus were identified on CT scan (Fig. 2a, b). After being diagnosed with BPF, the patient was referred to our department. It was considered that chest tube thoracostomy would not adequately treat the fistula and thoracic infection; thus, surgery was planned. In the operating room, we used a left-sided double-lumen tube for endobronchial intubation. After intubation, the bronchial fistula was evaluated by bronchoscopy and found to extend from the right main bronchus to the intermediate bronchus. Furthermore, ischemic changes were apparent around the fistula. Surgery was then performed via posterolateral thoracotomy. A fistula affecting the membranous portion of the right main bronchus and a little serous fluid around the fistula were noted (Fig. 3). The cause of necrosis and fistula appeared to be cauterization during esophagectomy. Because of the patient’s poor nutritional condition and the area of necrosis around the fistula, it was anticipated that simple covering of the fistula with an intercostal muscle flap would collapse. It was therefore considered necessary to devise a novel means of treating the fistula. First, a muscle flap was created by detaching the fourth and fifth intercostal muscles en bloc from the ribs near their angles (Fig. 4a), preserving blood flow from the right internal thoracic artery. Next, the muscle flap was passed between the azygos vein and right upper lobe bronchus (Fig. 4b, c) and the bronchus and pleural side of the intercostal muscle flap anastomosed using 4-0 monofilament synthetic absorbable suture material (Fig. 4d). The postoperative course was almost uneventful. On POD 1, we evaluated the post-surgical state of BPF by bronchoscopy. Since the muscle flap had closed BPF securely, the patient was extubated. Just for the next few days, he required nasal cannula oxygenation therapy and tracheal suctioning.

We performed intravenous antibiotic therapy after operation. As a result, the elevated C-reactive protein levels and white blood cell count gradually improved. In addition to that, there was no evidence of post-surgical empyema. Bronchoscopy was performed on POD 22. The bronchial lumen was patent without the muscle flap collapse. CT scan was performed on POD 30 after BPF closure. There were no findings of BPF and empyema, but it was difficult to diagnose the recurrence of esophageal cancer because the muscle flap and esophagectomy site overlapped. Although BPF improved, ADL of the patient did not improve due to depression. Therefore, he transferred from our hospital to a rehabilitation hospital on POD 76. After 4 months from our operation, he resulted in death due to aspiration pneumonia. However, according to the transferred hospital, there was no evidence indicating BPF and empyema on physical and chest X-ray findings.

## Discussion

Esophageal cancer is widely known as fatal and aggressive malignant disease. Therefore, treatment for esophageal cancer often needs multidisciplinary approach. However, patients with advanced esophageal cancer often suffer from malnutrition and treatment-related complications [6]. In this report, a patient who underwent esophagectomy suffered from BPF. BPF is a well-known complication of thoracic surgery [4], often being associated with empyema [7]. It has been reported that the rates of the post-esophagectomy complications of BPF and empyema are 0.3% and 2.3%, respectively [8]. Additionally, in this case, sarcopenia existed as a characteristic of esophageal cancer itself and one of the complications associated with preoperative treatment [6, 9]. Although pre- and postoperative nutritional care with parenteral and enteral nutrition was performed continuously, nutritional status hardly improved due to inflammation associated with cancer, thoracic infection, and multimodal treatment [9]. It was likely that the cause of BPF was not only intraoperative bronchial cauterization but also sarcopenia. The standard treatments for BPF and empyema associated with BPF are pleural drainage, irrigation, and surgery [10]. Post-esophagectomy BPF is difficult to treat because the patients are characteristically exhausted; thus, these cases usually require surgical management. Esophageal cancer-related respiratory tract fistulas often occur in the membranous portion of the trachea and main bronchus, as in the present case. Covering a membranous portion fistula with an intercostal muscle flap is an effective means of repair. The effectiveness of pedicled chest wall muscle flaps to close BPF was first reported by Abrashanoff in 1911 [11]. Fricke et al. reported that the advantage of reconstructive flap surgery is that it transfers well-vascularized tissue that may induce angiogenesis, thereby leading to improved wound healing and improved local efficacy of antibiotics [12]. The authors also mentioned that flaps should be sutured into the fistula without creating tension in the flap.

However, because of the patient’s exhaustion, minimizing the risk of muscle flap collapse is extremely important. We here report a novel procedure for creating an intercostal muscle flap and a route to the fistula. The essence of this procedure is the separation of the intercostal muscle flap from the dorsal side and subsequently passing it between the right upper lobe bronchus and the azygos vein. Even before suture is performed, the pedicled muscle flap wraps the bronchus from the ventral side to the dorsal side and thus naturally fits to the BPF on the dorsal side. Additionally, since the pedicle muscle flap passes through the cranial side of the right upper lobe bronchus, the right upper lobe bronchus maintains the muscle flap. As a result, closed BPF is free from the weight of the muscle flap. The azygos vein supports the muscle flap holding too. Not only strong fixing of the muscle flap and fistula, but the pedicle flap can also be made to function as a healing aid that maintains blood flow by the internal thoracic artery [5]. Even if the azygos vein is transected during esophagectomy, this procedure can be done by wrapping muscles above the bronchus if the location of the fistula is the same.

## Conclusion

In conclusion, we have herein reported a surgical procedure for treating BPF involving the right main bronchus in which an intercostal muscle flap is created and passed to the fistula via a novel route. We described what is important for BPF closure is not only the suturing technique but also the reduction of the slip risk and weight stress to the fistula. Our method is considered optimal to dorsal side BPF, especially in patients with poor nutritional condition.

## Data Availability

All datasets supporting the conclusions of this article are included in this published article.

## Abbreviations

BPF: Bronchopleural fistulas
BMI: Body mass index
UICC: Union for International Cancer Control
ADL: Activities of daily living
POD: Postoperative day
